# Efficacy of a bacterial 6-phytase supplemented beyond traditional dose levels on jejunal mucosa-associated microbiota, ileal nutrient digestibility, bone parameters, and intestinal health, and growth performance of nursery pigs

**DOI:** 10.1093/jas/skad134

**Published:** 2023-04-28

**Authors:** Vitor Hugo C Moita, Sung Woo Kim

**Affiliations:** Department of Animal Science, North Carolina State University, Raleigh, NC 27695, USA; Department of Animal Science, North Carolina State University, Raleigh, NC 27695, USA

**Keywords:** apparent ileal digestibility, bone breaking strength, intestinal health, growth performance, nursery pigs, phytase

## Abstract

This study aimed to determine the efficacy of a bacterial 6-phytase (*Buttiauxella spp.*) supplemented beyond traditional dose levels based on jejunal mucosa-associated microbiota, apparent ileal digestibility (AID), intestinal health and bone parameters, and growth performance of nursery pigs. Seventy-two weaned pigs (36 barrows and 36 gilts at 21 d of age with 5.8 ± 0.5 kg BW) were allotted to six treatments based on randomized complete block design with sex and initial BW as blocks and fed in three dietary phases (phase 1 for 14 d, phase 2 for 10 d, and phase 3 for 14 d). The treatments included a negative control (NC) diet without phytase formulated meeting nutrient requirements by NRC and the other five treatments were deficient in calcium (Ca) and phosphorus (P) by 0.12% with increasing levels of a bacterial 6-phytase (0, 500, 1,000, 2,000, and 5,000 FTU/kg feed). Titanium dioxide (0.4%) was added to phase 3 diets as an indigestible marker to measure AID of nutrients. On day 45, all pigs were euthanized to collect ileal digesta to measure AID, the third metacarpus to measure bone parameters, and jejunal mucosa to evaluate intestinal health and microbiota. Data were analyzed using the MIXED procedure for polynomial contrasts and the NLMIXED procedure for broken line analysis using the SAS 9.4. Broken line analysis demonstrated that 948 FTU/kg feed increased (*P* < 0.05) the ADG and the bone P content. Increasing phytase supplementation increased (linear, *P* < 0.05) AID of CP, bone P, and ash content. Increasing phytase supplementation reduced (*P* < 0.05) the fecal score during phases 2 and 3. Broken line analysis demonstrated that 1,889 FTU/kg feed increased (*P* < 0.05) bone breaking strength. Increasing phytase supplementation (PC vs. Phy) increased (*P* < 0.05) AID of ether extract (EE) and P. The supplementation of phytase at 2,000 FTU/kg feed tended (*P* = 0.087) to reduce the relative abundance of *Prevotellaceae*. In conclusion, the supplementation of a bacterial 6-phytase beyond traditional dose levels improved bone breaking strength, bone ash, and P content, AID of CP, EE, and P, and growth performance of nursery pigs with reduced relative abundance of Bacteroidetes specifically *Prevotellaceae* in the jejunal mucosa. Supplementation of a bacterial 6-phytase between 1,000 and 2,000 FTU/kg feed provided benefits associated with growth performance and bone parameters of nursery pigs.

## Teaser

Dietary supplementation of a bacterial 6-phytase between 1,000 and 2,000 FTU/kg feed provided benefits associated with changes in intestinal microbiota, apparent ileal digestibility of nutrients, bone parameters, and growth performance of nursery pigs.

## Introduction

Phytic acid has been described as an antinutritional factor because monogastric animals lack endogenous enzymes to degrade it. At a pH above 4, phytic acid would be bound to cationic minerals such as Ca, zinc (Zn), and copper (Cu) resulting in the formation of phytate complexes that are ­indigestible (Dersjant-Li et al., 2020). Phytic acid can also bind to AA and enzymes at pH levels above or below their isoelectric point, forming binary and ternary protein–complexes that are resistant to proteolytic enzyme hydrolysis (Deshpande and Cheryan, 1984).

Phytase, the enzyme responsible for catalyzing the hydrolysis of phytic acid, can be firstly classified based on their source used for production. Fungal phytases are commonly used in commercial diets for pigs, and they differ in regard to the microorganism used for production, optimum pH and temperature, and activity (Selle and Ravindran, 2008). Bacterial phytases tend to have a broader range for optimum pH (3.5 to 7.5) and temperature (37 to 70 °C) (Lei and Stahl, 2001), and to be resistant against proteolytic degradation by pepsin, trypsin, pancreatin, and papain when compared to fungal phytases (Simon and Igbasan, 2002).

Second, phytase can be classified based on the location where the hydrolysis of the inositol ring will initiate (Kumar et al., 2010). Generally, 3-phytases (EC 3.1.3.8) are characterized by initiating the dephosphorylation at the third carbon atom of the inositol ring, whereas the 6-phytases (EC 3.1.3.26) can initiate at the sixth carbon atom, which may result in increased dephosphorylation of the inositol ring (Cosgrove and Irving, 1980). Although 6-phytases may have higher efficacy dephosphorylating phytic acid and improving growth performance compared to 3-phytases (Moita et al., 2022a), there is no single phytase that can fully dephosphorylate it (Adeola and Cowieson, 2011).

The concept of supplementing phytase beyond traditional dose levels has been evaluated showing great potential for its implementation in diets for nursery and growing pigs (Lee et al., 2017; Holloway et al., 2019). The positive effects associated with supplementing phytase beyond traditional dose levels have been described as “extra-phosphoric” effects (Lu et al., 2019). A reduction of undigested phytic acid in the intestine may lead to changes in the intestinal environment, including a decrease in the pH which could favor the growth of beneficial bacteria over harmful ones (Zárate et al., 2002). Additionally, improving the hydrolysis of phytic acid increases the release of Ca and P, which was found to influence the mucosa-associated microbiota attachment in the mucosal layer of nursery pigs by the presence of divalent ions of Ca (Zárate et al., 2002).

Therefore, it was hypothesized that a bacterial 6-phytase supplemented beyond traditional dose levels can positively modulate the jejunal mucosa-associated microbiota and increase nutrient digestibility and enhance intestinal health and bone parameters leading to an increase in the growth performance of nursery pigs. The objective of this study was to investigate the efficacy of a bacterial 6-phytase supplemented beyond traditional dose levels on jejunal mucosa-associated microbiota, ileal nutrient digestibility, intestinal health and bone parameters, and growth performance of nursery pigs.

## Materials and Methods

The experimental protocol was approved by the Institutional Animal Care and Use Committee of North Carolina State University.

### Animal, design, and diets

The experiment was conducted at the Metabolism Educational Unit at North Carolina State University (Raleigh, NC). Seventy-two newly weaned pigs at 21 d of age (36 barrows and 36 gilts with initial BW of 5.8 ± 0.5 kg) were allotted to six dietary treatments based on a randomized complete block design with initial BW and sex as blocks. The six dietary treatments first consisted of an NC diet formulated to meet the requirements suggested by NRC (2012) without phytase supplementation. The other treatments consisted of a basal diet formulated with 0.12% deficient in Ca and P and split into five treatments including a positive control (PC) diet without phytase and four increasing levels of a bacterial 6-phytase (500, 1,000, 2,000, and 5,000 FTU/kg feed). Phytase (CJ Bio, Seoul, Korea) was premixed with corn and added to the treatment diets as shown in Table 1. The unit of phytase activity (FTU) was defined as the amount of phytase that liberates 1.0 μmol of inorganic phosphorus per minute at 40 °C and pH 5.5. The phytase activity analysis was performed using the Megazyme kit K-PHYTASE 09/21 (Megazyme, Bray Business Park, Bray, Co. Wicklow, A98 YV29, Ireland).

**Table 1.** Composition of experimental diets (as-fed basis)^1^

Item	Phase 1		Phase 2		Phase 3	
	NC	PC	NC	PC	NC	PC
Feedstuff, %						
Corn, yellow dent	49.87	50.67	56.04	56.94	62.95	63.80
Soybean meal, 48% CP	22.00	22.00	23.00	23.00	24.00	24.00
Whey permeate	15.00	15.00	10.00	10.00	5.00	5.00
Blood plasma	7.00	7.00	4.50	4.50	2.00	2.00
Poultry fat	1.50	1.50	2.00	2.00	1.50	1.50
L-Lys HCl	0.51	0.51	0.46	0.46	0.45	0.45
DL-Met	0.20	0.20	0.16	0.16	0.15	0.15
L-Thr	0.17	0.17	0.14	0.14	0.14	0.14
L-Val	0.07	0.07	0.05	0.05	0.03	0.03
L-His	0.01	0.01	0.00	0.00	0.00	0.00
L-Iso	0.07	0.07	0.02	0.02	0.00	0.00
Salt	0.22	0.22	0.22	0.22	0.22	0.22
Vitamin premix^2^	0.03	0.03	0.03	0.03	0.03	0.03
Trace mineral premix^3^	0.15	0.15	0.15	0.15	0.15	0.15
Dicalcium phosphate	0.80	0.00	0.90	0.00	0.85	0.00
Limestone	1.37	1.37	1.30	1.30	1.10	1.10
Titanium dioxide	0.00	0.00	0.00	0.00	0.40	0.40
Phytase premix^4^	1.00	1.00	1.00	1.00	1.00	1.00
BMD50^5^	0.03	0.03	0.03	0.03	0.03	0.03
Calculated composition						
DM, %	90.56	90.50	90.12	90.05	89.40	89.33
ME, kcal/kg	3,430	3,448	3,409	3,428	3,403	3,423
CP, %	23.76	23.81	21.87	21.91	21.00	21.05
SID^6^ Lys, %	1.50	1.50	1.35	1.35	1.23	1.23
SID Cys + Met, %	0.82	0.82	0.74	0.74	0.68	0.68
SID Trp, %	0.25	0.25	0.22	0.22	0.21	0.21
SID Thr, %	0.88	0.88	0.79	0.79	0.73	0.73
SID Val, %	0.95	0.95	0.86	0.86	0.81	0.81
SID Ile, %	0.79	0.79	0.75	0.75	0.75	0.75
SID Leu, %	1.64	1.64	1.56	1.57	1.52	1.52
Ca, %	0.85	0.73	0.80	0.68	0.70	0.58
STTD P^7^, %	0.45	0.33	0.40	0.28	0.33	0.21
Total P, %	0.70	0.57	0.65	0.51	0.58	0.44
Analyzed composition						
DM, %	89.56	89.35	88.53	88.52	88.36	88.34
CP, %	21.00	21.13	20.33	19.79	19.97	19.49
EE, %	4.08	4.06	4.56	4.52	4.14	4.15
NDF, %	6.30	6.46	7.92	7.45	8.02	7.04
ADF, %	2.67	2.81	3.03	3.34	3.44	3.00
Ca, %	1.02	0.86	0.83	0.70	0.74	0.61
Total P, %	0.67	0.51	0.60	0.46	0.58	0.43

^1^Diets in each phase were supplemented with increasing supplementation of 0, 500, 1,000, 2,000, and 5,000 FTU/kg of feed of a bacterial 6-phytase from (CJ BIO, Seoul, Korea). The analyzed phytase activity for the treatments NC, 0, 500, 1,000, 2,000, and 5,000 FTU/kg feed were 47, 68, 498, 1,021, 1,992, and 5,052 FTU/kg feed, respectively. One FTU of enzyme activity was defined as the amount of phytase that liberates 1.0 μmol of inorganic P per minute at 40 °C and pH 5.5. The phytase activity analysis was performed using the Megazyme kit K-PHYTASE 09/21 (Megazyme, Bray Business Park, Bray, Co. Wicklow, A98 YV29, Ireland).

^2^The vitamin premix provided per kilogram of complete diet: 6,614 IU of vitamin A as vitamin A acetate, 992 IU of vitamin D3, 19.8 IU of vitamin E, 2.64 mg of vitamin K as menadione sodium bisulfate, 0.03 mg of vitamin B12, 4.63 mg of riboflavin, 18.52 mg of D-pantothenic acid as calcium panthonate, 24.96 mg of niacin, and 0.07 mg of biotin.

^3^The trace mineral premix provided per kilogram of complete diet: 33 mg of Mn as manganous oxide, 110 mg of Fe as ferrous sulfate, 110 mg of Zn as zinc sulfate, 16.5 mg of Cu as copper sulfate, 0.30 mg of I as ethylenediamine dihydroiodide, and 0.30 mg of Se, as sodium selenite.

^4^Phytase enzyme mixed with corn.

^5^Bacitracin methylene disalicylate.

^6^SID = standardized ileal digestibility.

^7^STTD P = standardized total tract digestible phosphorus.

Pigs were individually housed in a pen and had free access to feed and water. The experimental period was 45 d, which was divided into three dietary phases: phase 1 (days 1 to 14), phase 2 (days 15 to 24), and phase 3 (days 25 to 45). The dietary phases were established according to the BW of the pigs. The BW and feed intake were recorded at the end of each week to calculate the average BW, ADG, ADFI, and G:F as indicators of growth performance. The fecal score was also recorded daily using a 1 to 5 scale: (1) very firm stool, (2) normal firm stool, (3) moderately loose stool, (4) loose, watery stool, and (5) very watery stool, as previously described (Cheng et al., 2021; 2022). In phase 3, TiO_2_ (0.4%) was added to the diets as an indigestible external marker to further determine the apparent ileal digestibility (AID) of nutrients.

### Sample collection and processing

After 45 d of feeding, all the pigs were euthanized to collect: the third metacarpus to measure bone parameters; mid-­jejunal mucosa (3 m after the pyloric duodenal junction; Cheng et al., 2021; 2022 to measure inflammatory and oxidative stress parameters and relative abundance and diversity of the mucosa-associated microbiota; and ileal digesta (a portion of 30 cm prior to the ileocecal valve) to measure the AID of nutrients. Mucosal samples from mid-jejunum were scraped, placed into 2 mL tubes, and later stored at −80 °C (after snap-freezing in liquid nitrogen, immediately after collection). The ileal digesta was collected into 150 mL containers and placed on ice, and then stored at −20 °C for measuring AID of DM, Ca, CP, EE, and P. The third metacarpus bone was separated and removed from all adhering soft tissue and cartilaginous end caps and stored at −20 °C for measuring bone breaking strength and mineral composition, and ash percentage.

One gram of frozen jejunal mucosa was taken with 2 mL phosphate-buffered saline solution (PBS) into 5 mL polypropylene tubes. Mucosa samples were ground on ice using a tissue homogenizer (Thermo Fisher Scientific, Waltham, MA) and transferred to new 2 mL micro-centrifuge tubes for centrifugation in 15 min at 14,000 × *g*. The supernatant was collected into eight sets of 0.5 mL polypropylene tubes and stored at −80 °C for further analysis. The sample collection and preparation procedures for analysis were performed as previously described (Moita et al., 2021b).

### Bone parameters

The bone breaking strength was measured right after collection and it was tested by using an axial servo-hydraulic load frame (858 Mini Bionix II, MTS Systems Inc., Minneapolis, MN). The instrument measures Newton’s (N) of force required to break the bones placed on two supports spaced 2.0 cm apart when force was applied to the center of the bone by an instrument moving at 30 mm/min. The breaking strength was measured by a pressure-sensitive cell and recorded on a graph recorder in N. After bone breaking strength assay the fat was removed from the bones using pure petroleum ether solution where bones were placed for 48 h. (Official Method 932.16, AOAC, 2006). Fat-extracted bones were dried for 24 h at 105 °C and then weighed and measured for length, and subsequently ashed at 600 °C for 24 h. Ashed samples were analyzed for the concentration of P by spectrophotometry (Official Method 946.06, AOAC, 2006) and Ca by flame atomic absorption spectroscopy (Official Method 968.08, AOAC, 2006). The bone processing and later analysis were performed as previously described by Babatunde et al., (2019) and Moita et al., (2021a).

### Apparent ileal digestibility

The frozen ileal digesta samples were dried by the freeze dryer (24D × 48, Virtis, Gardiner, NY). Dried digesta and feed samples were ground to fine powder form and stored in plastic containers for further analysis. Titanium dioxide concentration in the feed and digesta was measured as previously described by Moita et al. (2021b). The working range of the standards was 0 to 10 mg of TiO_2_. Samples were weighed around 0.5 g onto a tared weighing paper and then placed into 75 mL digestion tubes. One Kjeltab tablet (Fisher Scientific, Hampton, NH) and five pieces of selenized boiling granules were added to each digestion tube to prevent explosive vaporization. After adding 10 mL of concentrated H_2_SO_4_ (sulfuric acid), all digestion tubes were vortexed immediately. Then the tubes were heated for 2.5 h at 420 °C under a fume hood. When the tubes got cool after 30 min at room temperature, 2 mL of 30% H_2_O_2_ (hydrogen peroxide) was added to each tube 4 times and were vortexed until yellow to orange color appeared. Deionized water was added until reached the volumetric mark and then the tubes were covered and gently mixed. After that, 200 µL from the tubes were pipetted to a 96-well plate, which was read immediately at 410 nm. Titanium dioxide values were calculated based on the standard curve created from the concentration and absorbance of the respective standards.

The feed and digesta samples were weighed around 0.5 g to analyze the nitrogen content using TruSpec N Nitrogen Determinator (LECO CN-2000, LECO Corp., St. Joseph, MI) to later obtain the CP (6.25 × N). Furthermore, feed and digesta samples were weighed for determining DM (Method 934.01, AOAC, 2006), EE (Method 2003.06, .06, AOAC, 2006), P by spectrophotometry (Official Method 946.06, AOAC, 2006) and Ca by flame atomic absorption spectroscopy (Official Method 968.08, AOAC, 2006). The AID of DM, Ca, CP, EE, and P was calculated using the following equation as previously described (Passos et al., 2015; Chen et al., 2017; Moita et al., 2022b):


$$\begin{array}{ll} AID\;\left(\%\right)= & \;100\times \{1\left[(TiO_{2}feed/TiO_{2}digesta\right)\\ & \times \left(N\;digesta/N\;feed\right)]\} \end{array}$$

where “TiO_2_ feed” represents the titanium dioxide concentration in the feed, “TiO_2_ digesta” is the titanium dioxide concentration in the ileal digesta, “N feed” represents the nutrient concentration in the feed, and “N digesta” is the nutrient concentration in the ileal digesta.

### Inflammatory and oxidative stress parameters

Jejunal mucosa from three treatments was to be used for these parameters including NC, PC, and one level with phytase based on growth response and bone parameters. The concentrations of total protein, malondialdehyde (MDA), and protein carbonyl, were measured by the colorimetric method using commercially available kits according to instructions of the manufacturers. The absorbance was read using an ELISA plate reader (Synergy HT, BioTek Instruments, Winooski, VT) and software (Gen5 Data Analysis Software, BioTek Instruments, Winooski, VT). Mucosa samples were diluted (1:20) in working range of 0 to 2,000 μg/mL for the measurement of total protein using Pierce BCA Protein Assay Kit (#23225, Thermo Fisher Scientific). The amount of 25 μg/mL of each sample and standard was pipetted into a microplate well. The BCA working reagent (200 μg/mL) was added to each well and incubated at 37 °C for 30 min. Then, the absorbance was measured at 562 nm. The concentration was calculated based on the standard curve created from concentration and absorbance of the respective standard and further used to normalize the concentration of other parameters Duarte et al. (2019).

The TNF-α was measured following Porcine TNF-α Immunoassay Kit (#PTA00, R&D Systems; Minneapolis, MN). The working range of standards was 0 to 1,500 pg/mL and the absorbance was read at 450 and 550 nm. The IL-8 was measured following Porcine IL-8/CXCL8 Immunoassay Kit (#P8000, R&D Systems; Minneapolis, MN). For this analysis, mucosa samples were diluted (1:4) in a working range of 0 to 4,000 pg/mL and the absorbance was read at 450 and 550 nm. The concentrations of TNF-α and IL-8 were calculated based on the standard curve created from concentration and absorbance of the respective standard and described as pg/mg, following Holanda and Kim (2020).

Malondialdehyde was measured following OxiSelect TBARS MDA Quantitation Assay Kit (#STA-330, Cell Biolabs, San Diego, CA). The concentration range of MDA standards was 0 to 125 μM. The absorbance was measured at 540 nm. The concentration of MDA was calculated based on the standard curve created from concentration and absorbance of the respective standard and described as nmol/mg of protein, as previously described (Cheng et al., 2021; 2022.

Protein carbonyl was measured following OxiSelect Protein Carbonyl ELISA Kit (#STA-310, Cell Biolabs, San Diego, CA). All samples were diluted using PBS to reach the protein concentration of 10 μg/mL. The working range of standards was 0 to 7.5 nmol/mg protein. The absorbance was measured at 540 nm. The concentration of protein carbonyl was calculated based on the standard curve created from concentration and absorbance of the respective standard and described as nmol/mg of protein as previously described (Duarte et al., 2020).

### Relative abundance and diversity of the jejunal mucosa-associated microbiota

Jejunal mucosa from three treatments was to be used for these parameters including NC, PC, and one level with phytase based on growth response and bone parameters. The QIAamp Fast DNA Stool Mini kit (#51604, Qiagen; Germantown, MD) was used to perform the jejunal mucosa DNA extraction. The DNA extracted samples were sent to Mako Medical Laboratories (Raleigh, NC) for microbial sequencing using the 16S rRNA technique. The samples were prepared for the template using the Ion Chef instrument and sequenced using the Ion S5 system (Thermo Fisher Scientific). The Ion 16S Metagenomics Kit 113 (Thermo Fisher Scientific) was used to amplify the following variable regions of the 16S rRNA gene: V2, V3, V4, V6, V7, V8, and V9. The Torrent Suite Software (version 5.2.2; Thermo Fisher Scientific) was used to process and produce raw unaligned sequence data files of the relative abundance. To perform the sequence data analysis, alignment to GreenGenes and MicroSeq databases, alpha and beta diversity plot generation, and the operational taxonomic unit (OTU) table generation the Ion Reporter Software Suite (version 5.2.2) of bioinformatics analysis tools (Thermo Fisher Scientific) was utilized. Later, the Ion Reporter’s Metagenomics 16S workflow powered by QIIME (version w1.1) was used to analyze the samples. The relative abundances at phylum and family level were calculated based on the OTU data as previously described (Moita et al., 2022b). The “Others” were considered to represent the combined OTU with a relative abundance < 0.5%.

### Statistical analysis

Data were analyzed based on a randomized complete block design by the SAS 9.4 software (SAS Inc., Cary, NC, USA). Dietary treatments were considered fixed effects and the initial BW and sex blocks were considered random effects. Each treatment had 12 replicates (*n* = 12; and three body weight blocks within sex). The experimental unit was the pig, individually housed and fed. The analyses of growth performance, fecal score, nutrient digestibility, bone parameters, relative abundance, and diversity of the mucosa-associated microbiota in the jejunum and inflammatory and oxidative stress markers were performed using the MIXED procedure. To avoid biased data, six animals were removed from the study due to morbidity (1 from NC treatment, 1 from PC treatment, 2 from 500 FTU/kg feed treatment, and 2 from 1,000 FTU/kg). The linear and quadratic effects of increasing levels of phytase were tested by polynomial contrasts. Pre-planned contrasts were stablished to compare the inclusion of the phytase and no phytase inclusion diets (PC vs. Phy), negative control, and no phytase inclusion diets (NC vs. PC). When significant or tendency effects were found, the data were further analyzed using the NLMIXED procedure to determine the breakpoint to obtain the optimal phytase supplemental level, as previously described (Shen et al., 2012; Moita et al., 2021a). The predictor was set by multiplying the phytase inclusion level (FTU/kg feed) with the ADFI (0.633 kg/d) to account for the feed consumption of the animals through the experimental period (FTU/d). After the breakpoint was found, it was converted back from FTU/d to FTU/kg feed by dividing it with the ADFI (0.633 kg/d). For the broken-line model, the *P* value of each parameter indicates if the changes in the parameters are associated with the changes in the response. The relative abundance and diversity of the mucosa-associated microbiota in the jejunum, inflammatory, and oxidative stress parameter analysis were performed from three treatments: NC, PC, bacterial 6-phytase at 2,000 FTU/kg feed. Pre-planned contrasts were stablished to compare between negative control and positive control diets and between positive control diets and 2,000 FTU/kg diet. Statistical differences were considered significant with *P* < 0.05 and tendency with 0.05 ≤ *P* < 0.10.

## Results

### Growth performance

**Figure 1.**
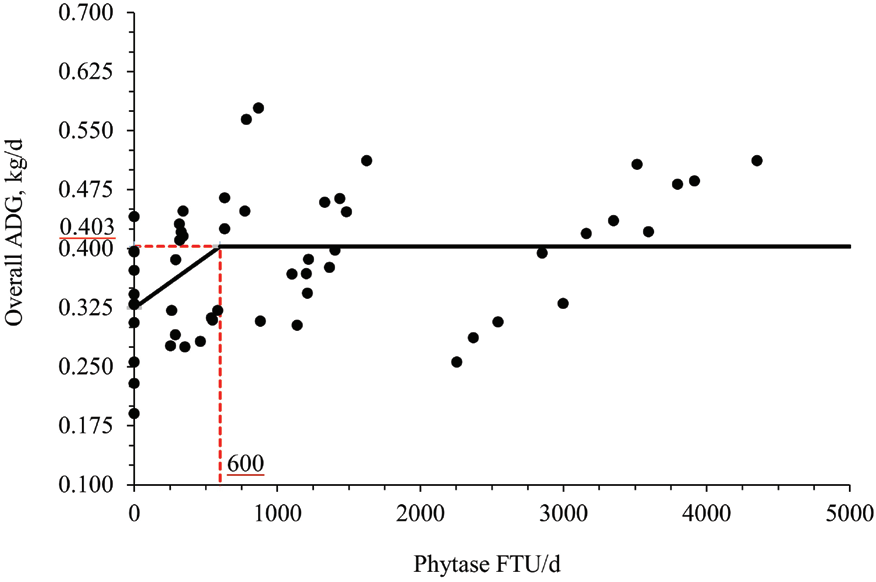
Changes in the overall ADG with supplementation of bacterial 6-phytase using a broken-line analysis. The break point was 600 ± 183 FTU/d of phytase supplementation when the overall ADG was 0.403 ± 0.014 kg/d. One slope broken-line model; the equation for overall ADG was *Y* = 0.403 − 0.00013 × *zl*; if phytase supplementation is ≥ breakpoint, then *z*; = 0; if phytase supplementation is < breakpoint, then *zl* = breakpoint − phytase supplementation. Values for phytase activity were based on the analyzed values. *P*-value for the plateau was < 0.001, for the slope was < 0.001, and for the breaking point was < 0.002. The breakpoint was converted from 600 FTU/d to 948 FTU/kg feed by dividing with the overall average feed intake (0.633 kg/d).

The BW of nursery pigs fed with NC diet was higher (*P* < 0.05) than pigs fed with PC diet at day 45 (Table 2). Phytase supplementation of (PC vs. Phy) increased (*P* < 0.05) the BW of nursery pigs at day 45. Increasing phytase supplementation tended to increase (linear, *P* = 0.064) the BW at day 45. The ADG of nursery pigs fed with NC diet was higher (*P* < 0.05) than pigs fed with PC diet during phase 3 and the overall period. Phytase supplementation (PC vs. Phy) increased (*P* < 0.05) the ADG of nursery pigs during phase 3 and the overall period. Increasing phytase supplementation increased (linear, *P* < 0.05) and had a quadratic effect (*P* < 0.05) (maximum: 698 g/d at 5,000 FTU/kg feed) on the ADG of nursery pigs during phase 3, and tended to increase (linear, *P* = 0.064) during the overall experimental period. The broken line analysis on the overall ADG of nursery pigs with different phytase supplemental levels indicated that the optimal phytase supplemental level is 600 FTU/d or 948 FTU/kg of feed (Figure 1).

**Table 2.** Growth performance of nursery pigs fed normal or calcium and phosphorus deficient diets with bacterial 6-phytase supplementation for 45 d

Item		Phytase, FTU/kg of feed						*P-*value			
	NC^1^	PC^2^	500	1,000	2,000	5,000	SEM	Linear	Quad^3^	NC vs. PC	PC vs. Phy^4^
BW, kg											
Initial BW	5.81	5.79	5.80	5.80	5.80	5.81	0.07	0.882	0.912	0.857	0.854
day 14	6.86	6.98	6.80	7.62	7.28	7.47	0.28	0.176	0.371	0.766	0.327
day 24	10.75	9.94	10.21	10.82	10.66	10.91	0.78	0.208	0.410	0.269	0.205
day 45	24.49	20.37	22.57	23.54	23.54	23.94	1.07	0.064	0.106	0.008	0.014
ADG, kg/d											
Phase 1	0.09	0.09	0.07	0.13	0.11	0.12	0.02	0.168	0.383	0.975	0.337
Phase 2	0.39	0.31	0.33	0.35	0.34	0.35	0.04	0.616	0.596	0.100	0.385
Phase 3	0.67	0.50	0.59	0.61	0.61	0.62	0.03	0.041	0.042	0.001	0.002
Overall	0.42	0.32	0.37	0.40	0.39	0.40	0.02	0.064	0.105	0.009	0.014
ADFI, kg/d											
Phase 1	0.14	0.16	0.14	0.22	0.19	0.18	0.02	0.497	0.123	0.454	0.419
Phase 2	0.67	0.62	0.57	0.75	0.68	0.68	0.06	0.394	0.270	0.523	0.439
Phase 3	1.01	0.78	0.90	0.95	0.95	0.94	0.05	0.091	0.030	0.001	0.005
Overall	0.68	0.58	0.62	0.67	0.65	0.65	0.03	0.266	0.091	0.016	0.045
G:F											
Phase 1	0.54	0.49	0.52	0.58	0.52	0.64	0.07	0.151	0.951	0.623	0.358
Phase 2	0.59	0.50	0.57	0.45	0.50	0.52	0.03	0.920	0.430	0.069	0.704
Phase 3	0.66	0.63	0.67	0.64	0.65	0.66	0.02	0.288	0.949	0.143	0.210
Overall	0.61	0.55	0.60	0.59	0.60	0.62	0.01	0.014	0.322	0.038	0.015

^1^Negative control.

^2^Positive control with 0 FTU/kg feed of phytase.

^3^Quadratic.

^4^Phytase supplementation. Six pigs were removed from the study with 1 from NC treatment, 1 from 0 FTU/kg feed treatment, 2 from 500 FTU/kg feed treatment, and 2 from 1,000 FTU/kg feed treatment due to morbidity (*n* = 66 total, *n* = 11 for NC, *n*=11 for PC, *n*=10 for the 500 FTU/kg of feed, *n* = 10 for the 1,000 FTU/kg of feed, *n*=12 for the 2,000 FTU/kg of feed, and *n*=12 for the 5,000 FTU/kg of feed).

The ADFI of nursery pigs fed with NC diet was higher (*P* < 0.05) than pigs fed with PC diet during phase 3 and the overall experimental period. Increasing phytase supplementation tended to increase (linear, *P* = 0.091) the ADFI during phase 3. Increasing phytase supplementation had a quadratic effect (*P* < 0.05) (maximum: 1,888 g/d at 5,000 FTU/kg feed) during phase 3 and tended to have a quadratic effect (*P* = 0.091) (maximum: 701 g/d at 4,500 FTU/kg feed) on the ADFI during the overall experimental period. Phytase supplementation (PC vs. Phy) increased (*P* < 0.05) the ADFI during phase 3 and the overall period.

The G:F of nursery pigs fed with NC diet was higher (*P* < 0.05) than pigs fed with PC diet during the overall experimental period and tended (*P* = 0.069) to be higher during phase 2. Increasing phytase supplementation improved (linear, *P* < 0.05) the G:F during the overall experimental period. Phytase supplementation (PC vs. Phy) increased (*P* < 0.05) the overall G:F of nursery pigs.

### Fecal score

The fecal score of nursery pigs fed with NC diet was lower (*P* < 0.05) than pigs fed with PC diet during phases 2 and 3 (Table 3). Increasing phytase supplementation decreased (linear, *P* < 0.05) the fecal score during phases 2 and 3. Phytase supplementation (PC vs. Phy) decreased (*P* < 0.05) the fecal score of nursery pigs during phases 2 and 3.

**Table 3.** Fecal score of nursery pigs fed normal or calcium and phosphorus deficient diets with bacterial 6-phytase supplementation for 45 d

Item	Phytase, FTU/kg of feed							*P-*value			
	NC^1^	PC^2^	500	1,000	2,000	5,000	SEM	Linear	Quad.^3^	NC vs. PC	PC vs. Phy^4^
*Fecal score* ^5^ *, %*											
Phase 1	3.31	3.33	3.20	3.38	3.16	3.36	0.11	0.695	0.347	0.884	0.628
Phase 2	3.03	3.49	3.14	3.22	3.17	3.06	0.09	0.023	0.214	0.002	0.004
Phase 3	3.07	3.25	3.10	3.12	3.13	3.02	0.05	0.021	0.556	0.030	0.019

^1^Negative control.

^2^Positive control with 0 FTU/kg feed of phytase.

^3^Quadratic.

^4^Phytase supplementation.

^5^The fecal score was recorded daily using a 1 to 5 scale: (1) very firm stool, (2) normal firm stool, (3) moderately loose stool, (4) loose, watery stool, and (5) very watery stool. Six pigs were removed from the study with 1 from NC treatment, 1 from 0 FTU/kg feed treatment, 2 from 500 FTU/kg feed treatment, and 2 from 1,000 FTU/kg feed treatment due to morbidity (*n* = 66 total, *n* = 11 for NC, *n* =11 for PC, *n* = 10 for the 500 FTU/kg of feed, *n* = 10 for the 1,000 FTU/kg of feed, *n* = 12 for the 2,000 FTU/kg of feed, and *n* = 12 for the 5,000 FTU/kg of feed).

### Bone parameters

**Figure 2.**
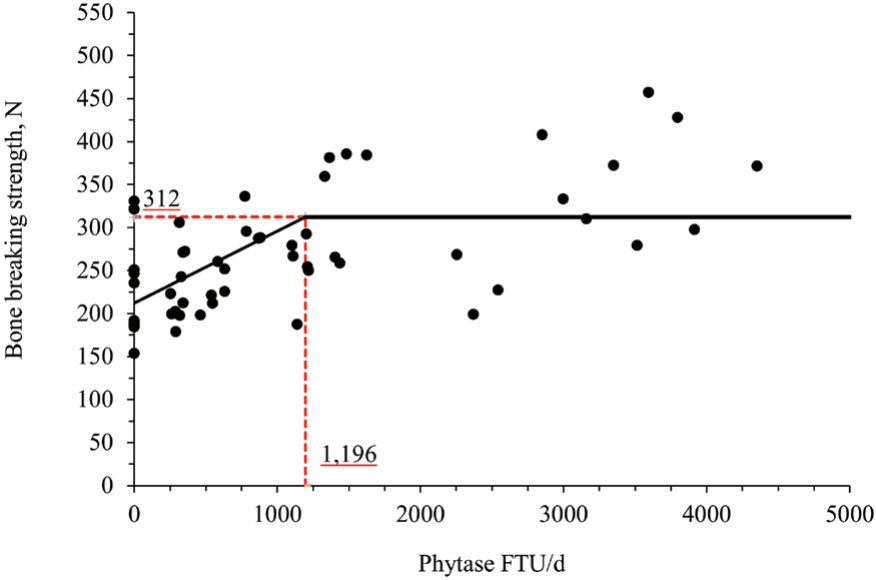
Changes in the third metacarpus bone breaking strength from nursery pigs at day 45 with supplementation of a bacterial 6-phytase using a broken-line analysis. The break point was 1,196 ± 230 FTU/d of feed of phytase supplementation when the bone breaking strength was 312 ± 12 N. One slope broken-line model; the equation for bone breaking strength was *Y* = 312 − 0.08393 × *zl*; if phytase supplementation is ≥ breakpoint, then *z*; = 0; if phytase supplementation is < breakpoint, then *zl* = breakpoint − phytase supplementation. Values for phytase activity were based on the analyzed values. *P*-value for the plateau was < 0.001, for the slope was < 0.001 and for the breaking point was < 0.001. The breakpoint was converted from 1,196 FTU/d to 1,889 FTU/kg feed by dividing with the overall average feed intake (0.633 kg/d).

The bone breaking strength of the third metacarpus of nursery pigs fed with NC diet was higher (*P* < 0.05) than pigs fed with PC diet (Table 4). Phytase supplementation (PC vs. Phy) increased (*P* < 0.05) the bone breaking strength of nursery pigs. Increasing phytase supplementation increased (linear, *P* < 0.05) the bone breaking strength of nursery pigs. The broken line analysis on the bone breaking strength of nursery pigs with different phytase supplemental levels indicated that the optimal phytase supplemental level is 1,196 FTU/d or 1,889 FTU/kg of feed (Figure 2).

**Table 4.** Bone parameters of the third metacarpus of nursery pigs at day 45 fed normal or calcium and phosphorus deficient diets with bacterial 6-phytase supplementation

Item	Phytase, FTU/kg of feed								*P-*value		
	NC^1^	PC^2^	500	1,000	2,000	5,000	SEM	Linear	Quad.^3^	NC vs. PC	PC vs. Phy^4^
Breaking strength, N	363.0	221.7	228.9	254.2	296.9	329.2	18.2	<0.001	0.146	<0.001	0.008
Metacarpus weight, g	9.2	8.1	8.2	8.8	9.0	8.9	0.4	0.130	0.107	0.049	0.166
Length, cm	5.3	5.1	5.1	5.2	5.2	5.3	0.1	0.110	0.165	0.111	0.155
DM^5^, %	62.1	56.6	60.5	57.6	62.2	62.0	2.7	0.241	0.410	0.159	0.199
Ca^6^, % of DM	11.1	9.7	9.8	10.7	10.0	10.6	0.3	0.099	0.586	0.002	0.112
P^7^, % of DM	5.4	4.6	4.7	5.2	4.9	5.2	0.1	0.024	0.409	<0.001	0.040
Ash, %	30.2	26.2	26.5	29.4	27.3	28.8	0.8	0.033	0.331	<0.001	0.038
Ca, % of ash	37.2	36.7	37.1	37.0	36.8	37.0	0.6	0.931	0.953	0.615	0.789
P, % of ash	17.9	17.6	17.8	17.9	18.0	18.1	0.2	0.080	0.338	0.170	0.085

^1^Negative control.

^2^Positive control with 0 FTU/kg feed of phytase.

^3^Quadratic.

^4^Phytase supplementation. Six pigs were removed from the study with 1 from NC treatment, 1 from 0 FTU/kg feed treatment, 2 from 500 FTU/kg feed treatment, and 2 from 1,000 FTU/kg feed treatment due to morbidity (*n* = 66 total, *n* = 11 for NC, *n* = 11 for PC, *n* = 10 for the 500 FTU/kg of feed, *n* = 10 for the 1,000 FTU/kg of feed, *n* = 12 for the 2,000 FTU/kg of feed, and *n* = 12 for the 5,000 FTU/kg of feed).

^5^Dry matter.

^6^Calcium.

^7^Phosphorus.

The metacarpus weight of the third metacarpus of nursery pigs fed with NC diet was higher (*P* < 0.05) than pigs fed with PC diet. The ash content in the bone of nursery pigs fed with NC diet was higher (*P* < 0.05) than of pigs fed with PC diet. Phytase supplementation (PC vs. Phy) increased (*P* < 0.05) the ash content in the bone of nursery pigs. Increasing phytase supplementation increased (linear, *P* < 0.05) the ash content in the bone.

**Figure 3.**
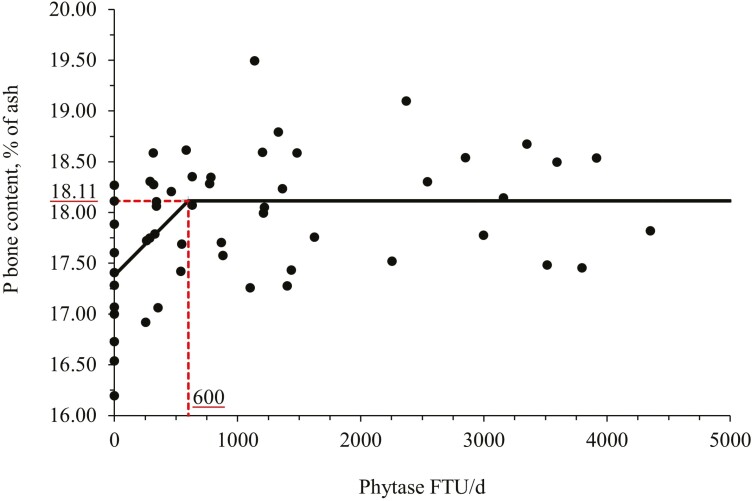
Changes in the P content in the third metacarpus bone based on the ash content of nursery pigs at day 45 with supplementation of a bacterial 6-phytase using a broken-line analysis. The break point was 600 ± 179 FTU/d of feed of phytase supplementation when the P bone content in the bone based on the ash content was 18.1 ± 0.1%. One slope broken-line model; the equation for P bone content was *Y* = 18.1 − 0.00122 × *zl*; if phytase supplementation is ≥ breakpoint, then *z*; = 0; if phytase supplementation is < breakpoint, then *zl* = breakpoint − phytase supplementation. Values for phytase activity were based on the analyzed values. *P*-value for the plateau was <0.001, for the slope was 0.010 and for the breaking point was 0.002. The breakpoint was converted from 600 FTU/d to 948 FTU/kg feed by dividing with the overall average feed intake (0.633 kg/d).

Increasing phytase supplementation tended to increase (linear, *P* = 0.080) the P concentration in the third metacarpus based on the ash content. Phytase supplementation (PC vs. Phy) tended to increase (*P* = 0.085) P content in the bone based on the ash content of nursery pigs. The broken line analysis on the P content in the bone based on the ash content of nursery pigs with different phytase supplemental levels indicated that the optimal phytase supplemental level is 600 FTU/d or 948 FTU/kg of feed (Figure 3). On the other hand, the Ca concentration in the bone based on the ash content was not affected by the treatments.

The Ca content in the third metacarpus based on the DM of nursery pigs fed with NC diet was higher (*P* < 0.05) than pigs fed with PC diet. Increasing phytase supplementation tended to increase (linear, *P* = 0.099) the Ca content based on DM. Moreover, the P content in the bone based on the DM of nursery pigs fed with NC diet was higher (*P* < 0.05) than pigs fed with PC diet. Phytase supplementation (PC vs. Phy) increased (*P* < 0.05) P content in the bone based on the DM of nursery pigs.

### Apparent ileal digestibility

Increasing phytase supplementation increased (linear, *P* < 0.05) the AID of CP (Table 5). Phytase supplementation (PC vs. Phy) increased (*P* < 0.05) the AID of EE. Increasing phytase supplementation tended to increase (linear, *P* = 0.055) the AID of P. The AID of P of nursery pigs fed with NC diet was higher (*P* < 0.05) than pigs fed with PC diet. Phytase supplementation (PC vs. Phy) tended to increase (*P* = 0.089) the AID of P.

**Table 5.** Apparent ileal digestibility of DM, CP, EE, Ca, and P of nursery pigs at day 45 fed normal or calcium and phosphorus deficient diets with bacterial 6-phytase supplementation

Item	Phytase, FTU/kg of feed								*P-*value		
	NC^1^	PC^2^	500	1,000	2,000	5,000	SEM	Linear	Quad.^3^	NC vs. PC	PC vs. Phy^4^
DM^5^, %	69.59	70.16	67.42	70.07	71.88	71.21	1.78	0.293	0.526	0.821	0.994
CP^6^, %	79.10	76.75	73.84	76.78	77.61	79.49	1.43	0.025	0.997	0.250	0.909
EE^7^, %	69.85	65.01	73.79	72.87	73.23	72.22	3.06	0.393	0.118	0.267	0.022
Ca^8^, %	72.74	70.86	74.16	74.52	75.92	74.69	2.31	0.399	0.188	0.568	0.131
P^9^, %	84.71	79.41	80.74	82.89	83.45	84.56	1.84	0.055	0.266	0.046	0.089

^1^Negative control.

^2^Positive control with 0 FTU/kg feed of phytase.

^3^Quadratic.

^4^Phytase supplementation; six pigs were removed from the study with 1 from NC treatment, 1 from 0 FTU/kg feed treatment, 2 from 500 FTU/kg feed treatment, and 2 from 1,000 FTU/kg feed treatment due to morbidity (*n* = 66 total, *n* = 11 for NC, *n* = 11 for PC, *n* = 10 for the 500 FTU/kg of feed, *n* = 10 for the 1,000 FTU/kg of feed, *n* = 12 for the 2,000 FTU/kg of feed, *n* = 12 for the 5,000 FTU/kg of feed).

^5^Dry matter.

^6^Crude protein.

^7^Ether extract.

^8^Calcium.

^9^Phosphorus.

### Inflammatory and oxidative stress parameters

The inflammatory and oxidative stress parameters in the jejunal mucosa of nursery pigs were not different between NC and PC, and between PC and phytase at 2,000 FTU/kg feed (Table 6).

**Table 6.** Oxidative stress and inflammatory parameters in the jejunal mucosa of nursery pigs at day 45 fed diets fed normal or calcium and phosphorus deficient diets with bacterial 6-phytase supplementation

Item		Phytase, FTU/kg of feed		SEM	*P*-value	
	NC^1^	PC^2^	2,000		NC vs. PC	PC vs. 2,000
MDA^3^, µmol/g of protein	0.53	0.57	0.55	0.04	0.544	0.718
PC^4^, nmol/mg of protein	2.69	3.04	2.61	0.31	0.370	0.375
IL-8^5^, pg/mg of protein	602	599	544	102	0.981	0.731
TNF-α^6^, pg/mg of protein	0.83	0.99	0.85	0.08	0.131	0.287

^1^Negative control.

^2^Positive control with 0 FTU/kg feed of phytase.

^3^Malondialdehyde.

^4^Protein carbonyl.

^5^Interleukin 8.

^6^Tumor necrosis factor alpha. Six pigs were removed from the study with 1 from NC treatment, 1 from 0 FTU/kg feed treatment, 2 from 500 FTU/kg feed treatment, and 2 from 1,000 FTU/kg feed treatment due to morbidity (*n* = 66 total, *n* = 11 for NC, *n*=11 for PC, *n*=10 for the 500 FTU/kg of feed, *n* = 10 for the 1,000 FTU/kg of feed, *n* = 12 for the 2,000 FTU/kg of feed, *n* = 12 for the 5,000 FTU/kg of feed).

### Relative abundance and diversity of the jejunal mucosa-associated microbiota

At the phylum level, the supplementation of phytase at 2,000 FTU/kg feed tended to reduce (*P* = 0.092) the relative abundance of Bacteroidetes when compared with PC diet (Table 7). No other differences were detected among the treatments at phylum level. At the family level, the supplementation of phytase at 2,000 FTU/kg feed tended to reduce (*P* = 0.087) the relative abundance of *Prevotellaceae* when compared with PC diet (Table 8). No other differences were detected among the treatments at family level.

**Table 7.** Relative abundance of the mucosa-associated microbiota in the jejunum at phylum level in nursery pigs at day 45 fed normal or calcium and phosphorus deficient diets with bacterial 6-phytase supplementation at 2,000 FTU/kg feed

Item		Phytase, FTU/kg of feed		SEM	*P-*value	
	NC^1^	PC^2^	2,000		NC vs. PC	PC vs. 2,000
Proteobacteria	44.55	44.04	58.79	15.65	0.978	0.439
Firmicutes	40.18	29.07	31.22	14.21	0.397	0.871
Bacteroidetes	11.23	15.45	1.80	5.26	0.581	0.092
Cyanobacteria	0.31	8.06	8.15	6.25	0.398	0.991
Others	2.33	1.42	0.04	1.17	0.536	0.361

^1^Negative control.

^2^Positive control with 0 FTU/kg feed of phytase. Six pigs were removed from the study with 1 from NC treatment, 1 from 0 FTU/kg feed treatment, 2 from 500 FTU/kg feed treatment, and 2 from 1,000 FTU/kg feed treatment due to morbidity (*n* = 66 total, *n* = 11 for NC, *n* = 11 for PC, *n* = 10 for the 500 FTU/kg of feed, *n* = 10 for the 1,000 FTU/kg of feed, *n* = 12 for the 2,000 FTU/kg of feed, *n* = 12 for the 5,000 FTU/kg of feed).

**Table 8.** Relative abundance of the mucosa-associated microbiota in the jejunum at family level in nursery pigs at day 45 fed normal or calcium and phosphorus deficient diets with bacterial 6-phytase supplementation at 2,000 FTU/kg feed

Item		Phytase, FTU/kg of feed		SEM	*P-*value	
	NC^1^	PC^2^	2,000		NC vs. PC	PC vs. 2,000
*Helicobacteraceae*	34.30	27.83	27.44	12.56	0.722	0.982
*Clostridiaceae*	26.68	15.74	15.80	6.79	0.245	0.737
*Veillonellaceae*	10.23	2.55	0.01	5.38	0.228	0.677
*Prevotellaceae*	8.58	13.97	1.75	4.63	0.427	0.087
*Succinivibrionaceae*	4.77	2.82	7.05	4.84	0.780	0.548
*Lachnospiraceae*	2.86	2.07	0.01	1.44	0.706	0.331
*Christensenellaceae*	1.48	4.92	0.00	2.05	0.279	0.131
*Lactobacillaceae*	1.45	0.01	10.52	6.89	0.806	0.213
*Caulobacteraceae*	1.29	3.10	17.61	8.04	0.876	0.226
*Enterobacteriaceae*	0.89	1.30	1.65	1.33	0.830	0.857
*Eubacteriaceae*	0.60	0.76	0.91	0.73	0.826	0.829
*Peptostreptococcaceae*	0.53	6.76	0.00	3.46	0.228	0.193
*Nostocaceae*	0.31	8.05	8.15	6.25	0.959	0.197
*Campylobacteraceae*	0.26	0.39	2.62	1.35	0.931	0.171
Others	9.04	10.95	3.50	6.65	0.669	0.120

^1^Negative control.

^2^Positive control with 0 FTU/kg feed of phytase. Six pigs were removed from the study with 1 from NC treatment, 1 from 0 FTU/kg feed treatment, 2 from 500 FTU/kg feed treatment, and 2 from 1,000 FTU/kg feed treatment due to morbidity (*n* = 66 total, *n* = 11 for NC, *n* = 11 for PC, *n* = 10 for the 500 FTU/kg of feed, *n* = 10 for the 1,000 FTU/kg of feed, *n*=12 for the 2,000 FTU/kg of feed, and *n* = 12 for the 5,000 FTU/kg of feed).

The alpha diversity of the mucosa-associated microbiota in the jejunum at the family level estimated with Chao1 (*P* = 0.050), Shannon (*P* = 0.062), and Simpson (*P* = 0.081) indexes from nursery pigs fed NC diet tended to be higher than pigs fed PC diet (Table 9). No differences were detected between nursery pigs fed PC diet and phytase at 2,000 FTU/kg feed.

**Table 9.** Alpha diversity of the mucosa-associated microbiota in the jejunum at family level in nursery pigs at day 45 fed normal or calcium and phosphorus deficient diets with bacterial 6-phytase supplementation at 2,000 FTU/kg feed

Item		Phytase, FTU/kg of feed		SEM	*P-*value	
	NC^1^	PC^2^	2,000		NC vs. PC	PC vs. 2,000
Chao1	4.84	2.82	3.50	0.66	0.050	0.477
Shannon	1.07	0.76	0.79	0.11	0.062	0.838
Simpson	0.62	0.46	0.47	0.08	0.081	0.956

^1^Negative control.

^2^Positive control with 0 FTU/kg feed of phytase. Six pigs were removed from the study with 1 from NC treatment, 1 from 0 FTU/kg feed treatment, 2 from 500 FTU/kg feed treatment, and 2 from 1,000 FTU/kg feed treatment due to morbidity (*n* = 66 total, *n* = 11 for NC, *n* = 11 for PC, *n* = 10 for the 500 FTU/kg of feed, *n* = 10 for the 1,000 FTU/kg of feed, *n* = 12 for the 2,000 FTU/kg of feed, and *n* = 12 for the 5,000 FTU/kg of feed).

## Discussion

Phytase supplementation in pig diets has been fairly well studied and provides benefits mainly associated with nutrient digestibility and bone parameters of nursery pigs and broiler chickens (Dilger et al., 2004; Moita et al., 2021a). Previous research has shown a variation among the results associated with growth performance, nutrient digestibility, and utilization between 3-phytases and 6-phytases, especially related to the growth stage when the animals were supplemented (Augspurger et al., 2003; Sands et al., 2009; Guggenbuhl et al., 2016; Cambra-López et al., 2020). This variation also extends to the comparison between fungal and bacterial phytases (Torrallardona et al., 2012; Almeida et al., 2013; Velayudhan et al., 2015; Dersjant-Li et al., 2020; Hardy et al., 2020). Bacterial phytases seem to work on broader pH and temperature range (Rodriguez et al., 2000), facilitating their use in different commercial conditions.

Recently, there was an increase in the practice of supplementing phytase beyond traditional dose levels, so-called “super-dosing” in pig diets aiming for extra-phosphoric effects (Lee et al., 2017). These effects are characterized by an “extra” release of nutrients, especially P (Lu et al., 2019) and *myo*-inositol (Walk et al., 2013) from phytate catalyzed by the inclusion of phytase 3-fold or greater than the recommended manufacturer dose (Holloway et al., 2019), which varies among different types of phytases. Inositol can be found primarily in mammalian tissues and cells as myo-inositol or phosphatidylinositol (Holub, 1986). According to Hawthorne and White (1976), phosphatidylinositol is considered a cellular mediator of signal transduction and regulates metabolism and growth.

In this study, the increasing supplementation of phytase enhanced the growth performance of nursery pigs by improving the overall ADG, ADFI, and G:F. The broken-line analysis indicated that the optimal supplemental level of phytase for overall ADG was 600 FTU/d or 948 FTU/kg feed. The results of this study agree with Lee et al. (2017) and Holloway et al. (2019), where the studies observed that the supplementation of phytase above 500 FTU/kg feed provided positive effects associated with mineral and protein utilization, ­phytate hydrolysis, and growth performance of nursery pigs. Moreover, Moran et al. (2017), observed that phytase supplemented at 2,500 FTU/kg provided the most benefits associated with growth performance of nursery pigs. On the other hand, during the growing and finishing phase the results showed more inconsistency. Holloway et al. (2019) and Langbein et al. (2013) reported no significant results when supplemented with phytase above traditional dose levels, whereas Braña et al. (2006) and Velayudhan et al. (2015) observed positive effects associated with growth performance and bone parameters with growing and finishing pigs. Overall, the supplementation of phytase beyond traditional dose levels seems to have more consistency when fed to nursery pigs and it can be partially explained due to the weaning stress that can impair the secretion of important endogenous enzymes resulting in negative effects on nutrient digestibility and consequently on the subsequent health and performance of the animals (Campbell et al., 2013; Moeser et al., 2017). Another possible reason is that when animals enter the growing-finishing phase, their digestive and immune system is matured, and it may not be seriously impacted throughout their productive life.

The main site of action of phytase is the stomach because under acidic pH phytase activity is enhanced and phytic acid is soluble which will facilitate its hydrolysis (Selle and Ravindran, 2008). When the digesta moves to small intestine the pH will change and phytic acid will bind to additional cationic compounds such as Ca, copper, and even AA becoming a salt (phytate) and insoluble (Angel et al., 2002). Thus, phytase should work in the stomach to effectively hydrolyze phytate considering its physical–chemical characteristics (Cowieson et al., 2016).

In this study, the increasing supplementation of phytase improved bone parameters such as bone breaking strength, ash, and P content on the bone. Additionally, the broken-line analysis indicated that the optimal supplemental level of phytase for bone breaking strength was 1,196 FTU/d or 1,889 FTU/kg feed and for P content in the bone was 600 FTU/d or 948 FTU/kg feed. The effects of phytase associated with improvements in bone parameters have been well investigated and it seems to be related to the greater uptake of P and Ca provided by the hydrolysis of the phytate and release of minerals that are important to support bone growth and mineralization of pigs (Braña et al., 2006; Varley et al., 2011). Improvements associated with the ash percentage in the bone may indicate an enhanced mineralization as a result of an increase in mineral availability due to the hydrolysis of the nutrient–phytate complexes (Sebastian et al., 1996).

According to Wu et al. (2018), commercial and experimental diets may have an excess of dietary Ca and underestimated P requirements due to factors such as formulation and mixing errors, neglecting mineral content in premixes and other additives, and variability of the feedstuffs used in those diets. An adequate balance between Ca and P is desired for optimal growth and bone mineralization. Even though Ca and P are being two of the most abundant minerals in the body (Oster et al., 2016), only P is present in a higher amount in the skeletal tissues (Nielsen, 1972). If either Ca or P levels are unbalanced, the bioavailability and utilization of both can be compromised (Létourneau-Montminy et al., 2015). Excess dietary Ca can impair P absorption in the small intestine by binding with phytate at neutral pH in the small intestine. As a consequence, it can reduce the release of P by phytase hydrolysis, regardless of the dietary P levels used in the diets (Heaney and Nordin, 2002; Dersjant‐Li et al., 2015). Thus, it is important to maintain balanced levels of Ca and P in order to not compromise bone growth and mineralization.

The results of this study show that phytase supplementation improved the AID of CP and EE, and tended to improve the AID of P. Phytic acid can also affect the digestion and absorption of proteins and fat (Helander et al., 1996; Selle et al., 2012). It will impact the AID of proteins and AA throughout the formation of binary and ternary protein–phytate complexes that will reduce their bioavailability. Negatively charged phytate will bind to positively charged protein molecules at a pH less than their isoelectric point (stomach pH), forming binary protein-phytate complexes. The binding will occur due to salt-like linkages that are formed between phytate and basic AA residues of lysine, arginine, and histidine (Cheryan and Rackis, 1980; Gilani et al., 2005). When the digesta moves to the small intestine, there will be a pH change toward to an increase in the isoelectric point of negatively charged proteins. As a result, there will be a formation of links between phytate and negatively charged proteins mediated by cationic bridges forming later protein–phytate ternary complexes (Cheryan and Rackis, 1980; Gilani et al., 2005). Additionally, phytate increases mucin secretion which can also be accounted as one of the negative effects on protein and AA digestibility (Cowieson et al., 2009). The results of AID of EE can be partially explained by the side activities of fiber and protein degrading enzymes that have their activity increased by phytate hydrolysis and the release of nutrients as well as enzymes (Helander et al., 1996; Kim et al., 2003; Liu et al. 2010). It has also been reported that phytate and inositol phosphate fractions can inhibit lipase activity (Knuckles, 1988), and reduce blood lipid concentration and hepatic lipogenesis in mice and rats (Szkudelski, 2005; Lee et al., 2007). Camden et al. (2001) reported a significant increase in the fat digestibility of starter broilers supplemented with phytase. Therefore, phytase may be a viable alternative to reduce the negative impacts of phytic acid on protein, AA, and EE digestibility, absorption, and utilization.

Regarding inflammatory and oxidative stress parameters, there is still no consensus about the positive effects of phytase over those parameters in nursery pigs. Although the samples were collected at 45 d of feeding to assess the long-term effects of phytase supplementation, it is important to note that inflammatory and oxidative stress responses may be more critical during earlier periods, such as upon the weaning. Karadas et al. (2005), reported that phytase supplementation can increase the hepatic concentration of the coenzyme Q_10_ (ubiquinone), which is a potent antioxidant mediator, in turkeys and broiler chickens. On the other hand, Karadas et al. (2009) observed that the greater uptake of nutrients through phytate hydrolysis, especially minerals, may increase the uptake of metal ions too, which may result in an increase the oxidative stress. Adequate P levels can be correlated to a positive modulation of the immune system (Kegley et al., 2001; Liu et al., 2008). Phosphorus is an important component of enzymes and different cell metabolites, such as lipopolysaccharides, phospholipids, and nucleic acids, that are potent mediators for the modulation of immune system and growth (Abelson, 1999; Hirota et al., 2010). Additionally, it may also affect the intestinal microbiota because P is a component of important co-enzymes and phospholipids in the cytoplasmic and outer membranes of gram-negative bacteria (Lengeler et al., 1999; Gresse et al., 2017).

Furthermore, Mann et al., (2014), found that high levels of Ca and P can influence the mucosa-associated microbiota attachment in the mucosal layer of nursery pigs by the presence of divalent ions of Ca (Zárate et al., 2002) and by steric hindrance of the mucosal binding sites. According to the authors, high levels of Ca and P can potentially increase the relative abundance of *Lactobacillus mucosae*, which is one the most abundant species in the mucus layer of the intestinal mucosa of pigs. Furthermore, in vitro studies also showed that free Ca ions may increase the mucosal adhesion of probiotic *Lactobacillus* strains (Larsen et al., 2007). These strains can effectively produce peptidic toxins, such as bacteriocin, and organic acids (Chauviere et al., 1992) that may inhibit the growth of important opportunistic bacteria, such as *Escherichia coli*, which is one of the bacterial strains associated with the postweaning diarrhea in pigs (Moeser et al., 2017; Duarte and Kim, 2021). Mann et al. (2014) also observed a decrease in the relative abundance of gram-negative bacteria, such as *Prevotella* and *Campylobacter*, which could be related to an increase in the competition for the mucosal binding sites in the intestinal mucosa between gram-positive and gram-negative bacteria. These findings agree with the results from this study where a bacterial 6-phytase supplemented at 2,000 FTU/kg feed reduced the relative abundance of *Prevotellaceae*, which is one of the major gram-negative bacteria found in the jejunal mucosa (Duarte and Kim, 2021; 2022).

As earlier described, phytase supplementation aims to increase the hydrolysis of phytic acid and the release of important nutrients, especially Ca and P. The extra phosphoric effects, reported as one of the benefits of supplementing phytase beyond the traditional level also describe an increase in the breakdown of phytate–mineral complexes as one of those effects (Walk et al., 2013). With a greater uptake of minerals, especially Ca and P, a positive modulation of the mucosa-associated microbiota by increasing the relative abundance of *Lactobacillus* strains may be expected. A study conducted by Moita et al. (2021a) showed a tendency on the increase of the relative abundance of *Lactobacillus* in the jejunal mucosa when phytase was supplemented at 2,000 FTU/kg of feed in broiler chickens. This could be due to a greater uptake of Ca and P possibly caused by the high dosing levels of phytase supplemented in the diets (Lu et al., 2019). On the other hand, there was no influence of phytase on changes in oxidative damages to the jejunal mucosa of broiler chickens, which contrasts with other studies showing positive effects on the intestinal health and microbiota in broiler chickens and nursery pigs when phytase was supplemented beyond its traditional levels (Liu et al., 2008; Lu et al., 2019).

Previous studies reported positive effects on the intestinal health of nursery pigs by “super dosing” corn-expressed phytase. Lee et al. (2016) observed improved growth performance and villus height in the duodenum and a tendency for the reduction of TNF-α and MDA concentrations in the duodenum and jejunum. In another nursery study conducted by Shili et al. (2020), it was observed improvements in the growth performance, bone mineralization, and high relative abundance of fecal Actinobacteria and *Bifidobacterium* of nursery pigs when corn-expressed phytase was supplemented at 2,000 FTU/kg feed and 4,000 FTU/kg. Although in this study the supplementation of bacterial-6 phytase did not show significant effects on the inflammatory and oxidative status parameters, more research should be conducted to better elucidate the mechanism that phytase can positively affect the inflammatory and oxidative stress of pigs.

The supplementation of phytase can provide different benefits associated with growth performance, bone parameters, and nutrient digestibility. However, recent studies are indicating that the supplementation of phytase beyond traditional dose levels may express extra-phosphoric effects by increasing the hydrolysis of phytate and generation of greater levels of *myo*-inositol. Extra-phosphoric effects may positively affect not only the growth performance and nutrient digestibility but also bone and intestinal health parameters of pigs. In conclusion, the supplementation of a bacterial 6-phytase beyond traditional dose levels improved bone breaking strength, bone ash, and P content, AID of CP, EE, and P, and growth performance of nursery pigs with the reduced relative abundance of Bacteroidetes specifically *Prevotellaceae* in the jejunal mucosa. Supplementation of a bacterial 6-phytase between 1,000 and 2,000 FTU/kg feed provided benefits associated with growth performance and bone parameters of nursery pigs.

## Abbreviations:

ADF: acid detergent fiber
ADFI: average daily feed intake
ADG: average daily gain
AID: apparent ileal digestibility
BW: body weight
DDGS: distillers’ dried grains with soluble
Ca: calcium
CP: crude protein
Cu: copper
DM: dry matter
FTU: phytase unit
EE: ether extract
ELISA: enzyme linked immunosorbent assay
G:F: gain to feed ratio
IL-8: interleukin 8
MDA: malondialdehyde
N: nitrogen
NC: negative control
NDF: neutral detergent fiber
OTU: operational taxonomic unit
P: phosphorus
PBS: phosphate-buffered saline solution
PC: positive control
Phy: phytase
TiO_2_: titanium dioxide
TNF-α: tumor necrosis factor alpha
Zn: zinc
